# Mitochondria-Targeted Antioxidants, an Innovative Class of Antioxidant Compounds for Neurodegenerative Diseases: Perspectives and Limitations

**DOI:** 10.3390/ijms24043739

**Published:** 2023-02-13

**Authors:** Matteo Fields, Annalisa Marcuzzi, Arianna Gonelli, Claudio Celeghini, Natalia Maximova, Erika Rimondi

**Affiliations:** 1 Department of Translational Medicine, University of Ferrara, 44121 Ferrara, Italy; 2 Department of Environmental and Prevention Sciences, University of Ferrara, 44121 Ferrara, Italy; 3 Department of Pediatrics, Pediatrics, Bone Marrow Transplant Unit, Institute for Maternal and Child Health-IRCCS Burlo Garofolo, 34137 Trieste, Italy; 4 Department of Translational Medicine and LTTA Centre, University of Ferrara, 44121 Ferrara, Italy

**Keywords:** neuroinflammation, mitochondria, mitophagy, neurodegenerative disorders, mitochondrial medicine

## Abstract

Neurodegenerative diseases comprise a wide spectrum of pathologies characterized by progressive loss of neuronal functions and structures. Despite having different genetic backgrounds and etiology, in recent years, many studies have highlighted a point of convergence in the mechanisms leading to neurodegeneration: mitochondrial dysfunction and oxidative stress have been observed in different pathologies, and their detrimental effects on neurons contribute to the exacerbation of the pathological phenotype at various degrees. In this context, increasing relevance has been acquired by antioxidant therapies, with the purpose of restoring mitochondrial functions in order to revert the neuronal damage. However, conventional antioxidants were not able to specifically accumulate in diseased mitochondria, often eliciting harmful effects on the whole body. In the last decades, novel, precise, mitochondria-targeted antioxidant (MTA) compounds have been developed and studied, both in vitro and in vivo, to address the need to counter the oxidative stress in mitochondria and restore the energy supply and membrane potentials in neurons. In this review, we focus on the activity and therapeutic perspectives of MitoQ, SkQ1, MitoVitE and MitoTEMPO, the most studied compounds belonging to the class of MTA conjugated to lipophilic cations, in order to reach the mitochondrial compartment.

## 1. Introduction

Neurodegenerative diseases elicit the progressive degeneration or death of neural cells, provoking either movement disorders, such as in ataxias, Amyotrophic Lateral Sclerosis (ALS) and Multiple Sclerosis (MS), or cognitive impairments (dementias), such as Alzheimer’s Disease (AD) and Parkinson’s Disease (PD). These are pathologies with increasing incidence in developed countries, accompanying a progressive aging of the population [1,2,3].

In this context, Stanga et al. [4] determined that mitochondrial dysfunctions represent a crucial point of convergence in the pathogenesis of such neurodegenerative pathologies.

Recent studies, indeed, have highlighted the role of mitochondria not only in normal brain functions but also in the pathogenesis of its diseases: the main actors in these conditions are mitochondrial dysfunction and excessive oxidative stress, which are often found as linked events [5,6]. Neuronal transmission, integrity and survival all depend on efficient mitochondria, responsible for ATP production (required for the transport and release of neurotransmitters) and contributing to calcium homeostasis and apoptosis control [5,7]. At the same time, the oxidative phosphorylation leading to ATP generation also produces free radicals, namely reactive oxygen species (ROS), that make mitochondria susceptible to oxidative stress. Therefore, even minor alterations of the redox equilibrium induce ROS accumulation and mitochondrial dysfunction, accompanied by severe consequences, primarily energetic failure, excitotoxicity and rupture of the mitochondrial membrane [5,7,8].

### 1.1. Mitochondrial Dynamics

**Figure 1:**
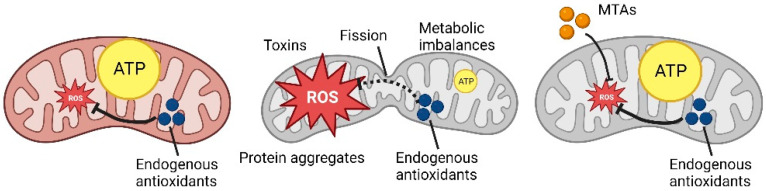
Schematic representation of ROS production and MTAs mechanism (Created with Biorender.com).

However, mitochondria are extremely plastic organelles, and the endogenous antioxidant system (involving glutathione and other reducing agents) is not the only coping mechanism that a cell can use (Figure 1).

Mitochondria’s structure can be rearranged through two important processes [5,9]: mitochondrial fission, the division of a mitochondrion into two separate mitochondria, an event that generates a fragmented mitochondrial network with several rounded mitochondria; contrarily, mitochondria fusion is the merging of two or more mitochondria that generates a hyperfused network with elongated mitochondria highly connected with one another. Mitochondrial dynamics—that is, the balance between the fission and fusion mechanisms—represents the pivotal point in mitochondrial and, more broadly, cellular homeostasis [9,10]. In fact, we must consider that the main purpose of this equilibrium is not limited to the maintenance of mitochondrial function; it is also crucial to guarantee an adequate energetic and metabolic supply to the cell [11,12]. Morphologic changes at this level represent a cellular response to specific physio-pathologic conditions that require a considerable energetic consumption from the very same mitochondria [13,14].

In non-physiologic conditions, an external stimulus elicits an adaptive response that translates into the remodeling of mitochondria, which may induce a partial or complete blockade of their motility or, on the other hand, the fragmentation of their network. Specifically, this last event is characterized by a condition of high ROS production that determines oxidative stress and cell death, in turn triggering an increased mitochondrial fission [15,16]. In this scenario, the fragmentation might be useful to prevent the damage caused by ROS and to facilitate the elimination of damaged mitochondria. On the contrary, an excessive mitochondrial fusion seems to be associated with a pronounced mitochondrial elongation that confers protection from the autophagy mechanism, inhibiting the formation of phagosomes [17,18].

The process of mitochondria degradation, called mitophagy, is finely regulated [19,20,21,22]: PINK1 (PTEN Induced Kinase 1 or PARK6), a protein kinase, translocates on the outer membrane of a damaged mitochondrion that loses physiological membrane potential. Parkin (or PARK2), a ubiquitin ligase, is recruited and activated by PINK1, leading to ubiquitination of the mitochondrion, which, in this way, is engulfed by a phagosome and degraded when the phagosome fuses with a lysosome. This process is activated to get rid of fragmented mitochondria and recycle their components [20,23], destined to be the biogenesis of new functional mitochondria, and is particularly critical for highly demanding cells such as neurons that need to keep under control the energy supply and redox status, thus relying on the efficient elimination and replacement of dysfunctional mitochondria with a high ROS content [24,25,26].

Generally speaking, ROS mostly derive from the mechanism of energy production taking place in the mitochondria, and they are countered by the cellular antioxidant system [27,28]. Indeed, the oxidative stress is represented by an imbalance between the concentration of generated ROS and the antioxidant potential that counters them, determining an alteration of the intracellular signaling and redox status. We can observe a wide spectrum of adaptive cellular responses as the oxidative stress increases: in fact, a low level determines changes in genes and protein expression with no structural changes in the mitochondria, while a significant level can lead to high fragmentation and eventually cell death [29,30].

### 1.2. Neurodegenerative Disorders Characterized by Mitochondrial Involvement

In the past 30 years, an increasing body of evidence has pointed at the role of mitochondria in the brain homeostasis, as well as the pathogenesis of neurodegenerative diseases, in which they may have a main role or be involved in a second moment exacerbating the neuronal damage [31] (Table 1).

Parkinson’s Disease is a progressive motor disorder, affecting 1% of the world population over the age of 60 years, caused by neurodegeneration occurring in the substantia nigra [32,33,34]. Symptoms comprise the characteristic resting tremor and postural instability, muscle rigidity, bradykinesia and, in some cases, sleeping disorders and cognitive impairment. The nigral dopaminergic neurons are the major cell type involved in the disease, due to their high reliance on oxidative phosphorylation, determining their susceptibility to oxidative stress [32,33]. Moreover, in PD patients, this brain region shows an accumulation of intraneuronal Lewy bodies that retain aggregated proteins, mainly α-synuclein but also neurofilaments, ubiquitin and others [33,34,35]. Physiologically, α-synuclein seems to be implicated in the transport and release of neurotransmitters; however, when aggregates are formed, they can interact with transport machineries present on the outer mitochondrial membrane, blocking their activity, with negative consequences on mitochondrial functions, primarily oxidative phosphorylation, followed by ROS accumulation, alteration of the membrane potential and impaired calcium homeostasis [33,34]. This process was confirmed both in vivo, where mutant α-synuclein potentiated the effects of PD-inducing toxins in a mouse model [36], and in patients’ biopsies [37]. Moreover, α-synuclein can be released in the extracellular space and taken up by nearby neurons, where it induces protein aggregation, spreading the formation of Lewy bodies. Other evidence shows a correlation between mitochondrial dysfunctions and α-synuclein aggregation: cases of MPTP (1-methyl-4-phenyl-1,2,3,6-tetrahydropyridine) intoxication [38] and studies on animals exposed to MPTP [39] and herbicides (paraquat and rotenone) [40,41,42] reported the inhibition of Complex I of the respiratory chain, with the consequent accumulation of ROS and aggregates, even if the process leading to the latter effect still needs in-depth studies. Cases of familial PD, instead, are linked to alterations in the process of mitophagy: mutations in the gene PINK1 and Parkin were identified as drivers of PD, since the impaired degradation of dysfunctional mitochondria leads to neurodegeneration [33,34].

Alzheimer’s Disease is an age-related neurological disease, caused by the progressive degeneration of cognitive functions, starting with memory loss up to the loss of ability to perform daily tasks. These problems are caused by the progressive accumulation of amyloid β (Aβ) both in the extracellular milieu and within the cell and the formation of hyperphosphorylated tau-protein tangles [32,34,43,44]. The link between Aβ aggregation and mitochondrial dysfunction has been demonstrated, and some studies have revealed a bidirectional relationship. It was proposed that mitochondrial dysfunction and altered metabolism were early features of AD, a hypothesis supported by different studies [32,34,43,45,46] revealing alterations of the metabolism independent of Aβ that caused the formation of Aβ aggregates and tau fibrils, the latter being a consequence of tau protein hyperphosphorylation. Mitochondrial defects and oxidative stress may result from age, sedentary lifestyle, poor exercise and a high-calorie diet [43,45]. At the same time, in other studies, researchers observed that exposure to Aβ aggregates was the driving force of mitochondrial damage, impaired calcium homeostasis, ROS overproduction, oxidative stress and energy shortage. Fibrils of tau protein also contributed to respiratory chain impairment [32,34,43,44,45]. Ultimately, both Aβ and tau aggregates interfere with mitochondria trafficking, blocking their transport towards synapses; mitochondrial dynamics and biogenesis, shifting the equilibrium towards fission and generating small fragmented mitochondria that cannot be replaced by new mitochondria; and mitophagy, blocking the signaling molecules mediating the fusion with autophagosomes/lysosomes [32,34,43,44,45,46].

Amyotrophic Lateral Sclerosis is characterized by progressive muscle paralysis mediated by damage to the upper and lower motor neurons, causing severe debilitation and later death within a few years after the diagnosis [47,48,49,50]. Genetic mutations responsible for the pathogenesis are still under investigation, with few confirmed as causative mutations. Despite the genetic variability, the common hallmarks of ALS are the mitochondrial and endoplasmic reticulum abnormalities, as well as the disruption of the signaling between these compartments [47,48,49,50]. Mitochondria dysfunctions are caused by mutated proteins that can either form aggregates or stimulate mitochondria fragmentation. Another characteristic of ALS is the presence of aggregated, swollen and vacuolated mitochondria [47,48,49,50]. Therefore, mitochondria become dysfunctional, showing impaired ATP production and ROS removal, leading to progressive neuron death, which exacerbates the pathological condition.

Multiple Sclerosis is an autoimmune disease that causes muscular weakness, ataxia, cognitive impairments and autosomal nervous system dysfunction. The main driving force is the autoreactivity of CD4+ T-helper type 1 cells against myelin components [51,52,53]. When immune cells cross the blood–brain barrier and reach the CNS, they provoke demyelination and inflammation, with profound deleterious effects on neurons. Myelin sheath loss hampers the action potential, because the exposure of more axonal membrane portions slows down the signal transmission and increases the energetic demand to activate the membrane ion channels [54]. Consequently, the mitochondrial respiratory chain is overactivated to supply sufficient ATP, causing the accumulation of ROS and exhaustion of antioxidant systems, with the contribution of tissue inflammation [51,52,53,55]. ROS are responsible for damages to mitochondrial membranes and mtDNA, lipid peroxidation and mitochondrial fragmentation, impairing the already worn out electron transport chain, with consequent decreased ATP production, which worsens the action potential transmission [51,52,53,55].

Huntington’s Disease is an autosomal-dominant disease involving progressive neurodegeneration that affects 5–10 per 100,000 individuals in Western countries. The genetic basis of this disease is a copy number variation of a CAG (cytosine–adenine–guanine) repeat in the huntingtin gene (HTT), causing expansion of the repeats that generates a mutant HTT protein with a longer N-terminal polyglutamine sequence [19,56,57,58]. Affected neurons are mostly found in the striatum, causing tissue damage and atrophy; however, the damage can spread to cortical areas and other regions. HTT mutation leads to protein accumulation and aggregation in the cytoplasm and nucleus, where it interferes with other proteins, impairing normal neuronal functions [19,58]. Normally, the protein participates in different trafficking mechanisms, including axonal transport, nuclear transport, autophagy and mitophagy, in which it interacts with and stabilizes different protein complexes [19,58]. Alterations of its activity lead to oxidative stress, mitochondrial dysfunction and excitotoxicity. In fact, in the striatum of mouse models [59] and human biopsies [60,61] of HD, researchers found higher levels of ROS than in control subjects, associated with reduced or no activity of Complexes II, III and IV of the respiratory chain. HTT aggregates also induce increased mitochondrial fission, reducing the energetic efficiency with a consequent ROS overproduction [56,62]. This is exacerbated by impaired mitochondria trafficking and mitophagy, which cause the accumulation of dysfunctional mitochondria at synapses that, in turn, start degenerating because of the lack of ATP and neurotransmitter release [19,56,63].

## 2. Role of Antioxidants on Mitochondrial Homeostasis

**Figure 2:**
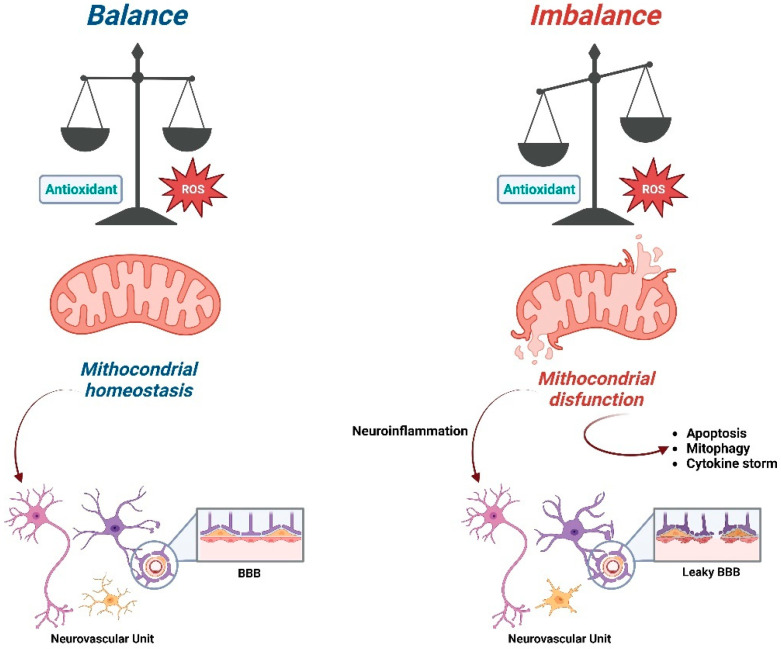
Schematic representation of the consequences of the imbalance ratio between ROS and antioxidant compounds on mitochondrial functions (Created with Biorender.com).

Studies have largely shown and characterized the importance and properties of antioxidants as molecules able to counter the detrimental effects of free radicals, thus protecting cellular integrity [64]. These molecules prevent or at least delay oxidation by the removal or inhibition of oxidizing agents, and secondarily, they contribute to interrupt the harmful oxidation after its initiation by transforming free radicals in nonreactive compounds. The role of antioxidant molecules is crucial in the therapeutic strategies for neurodegenerative diseases [65,66] (Figure 2).

The brain and neurons are characterized by a high metabolic demand, especially in terms of energy consumption [6]. However, they also present low levels of antioxidant molecules that make them prone to dangerous ROS accumulation and oxidative damage, favoring cellular and tissue damages, neuroinflammation and blood–brain barrier permeability. Given this evidence, it is suggested that defective mitochondrial activity plays a pivotal role in the pathogenesis of neurodegenerative diseases, including Alzheimer’s Disease, Parkinson’s disease, Amyotrophic Lateral Sclerosis, Multiple Sclerosis and Huntington’s Disease [6,67].

These are severely impairing diseases affecting millions of people worldwide, limiting their quality of life and life expectancy. Studies about the physiology of the nervous system have shown that the imbalance in energy metabolism could be a target for new treatment strategies in neurodegenerative diseases and that the synergy between activity and metabolic pathways should be considered [68,69]. For this reason, many molecules have been investigated to assess their effects on mitochondria in oxidative stress conditions.

Many studies have been carried out to test the potentialities of conventional antioxidants to protect from or revert the oxidative damage, stabilizing the physiological condition of the mitochondria. Despite promising results obtained in in vitro and in vivo experiments on cellular and animal models, these molecules failed to deliver beneficial effects when administered in clinical trials [70,71].

Few hypotheses were formulated to explain their underwhelming performances. First of all, being nontargeted molecules, these antioxidants could accumulate in different organs, reducing the amount available in the target tissue [72]. It was also noted that excessive or inappropriate antioxidant administration could induce a complete shutdown of ROS production, resulting in the upregulation of mitogen-activated protein kinase (MAPK) pathways as compensatory mechanisms [73,74] or in the disruption of essential signaling pathways important for the cells and the organism [72]. In this way, the drugs contrast with the endogenous antioxidant system, with a negative impact on the cells. Secondly, experimental factors might have led to unsatisfying results, such as inefficient administration, a small number of recruited patients, the choice of a trial’s endpoints or an advanced stage of the disease at the time of drug administration [70,71].

Investigators were also concerned by the bioavailability of nontargeted antioxidants, because they could not determine if they were properly absorbed and metabolized not only in the target tissue but also in the rest of the body [70,72]. These uncertainties raised questions about the adequate dose to administer to achieve a therapeutic effect in neurodegenerative diseases.

A possible and promising solution seems to be the conjugation of antioxidant molecules with a carrier, developing a targeted treatment for diseased tissues with minor off-target effects. Among those available, researchers’ efforts were focused on lipophilic cations, liposomes and peptides, aimed at increasing the penetration into dysfunctional mitochondria to restore their activity and the redox status [70,74]. Targeted delivery to these organelles makes it easier to obtain a higher concentration where needed the most, protecting cells and tissues from oxidative damage.

## 3. Mitochondria-Targeted Antioxidants (MTAs), Innovative Antioxidants Drugs

In this review, we chose to present in detail only the class of mitochondria-targeted antioxidants conjugated to lipophilic cations.

Among the possible carriers, triphenylphosphonium (TPP) has been largely studied and used for the transport of antioxidant molecules to mitochondria, especially the hydrophobic ones that have difficulties in passing through cellular membranes to reach the cytoplasm and then the mitochondrial compartment [70].

TPP, similar to the other lipophilic cations, can be used for targeted delivery to mitochondria due to its positive charge, attracted by the high mitochondrial transmembrane potential. In fact, the electrochemical gradient found between the cellular membrane (−30 to −60 mV) and the inner mitochondrial membrane (−150 to −180 mV) provides an incredible driving force for the accumulation of lipophilic cations into mitochondria. This negative membrane potential is unique to mitochondria, making lipophilic cations highly selective for this subcellular compartment and avoiding unspecific accumulation in others [71,75,76]. Therefore, after entering the cell exploiting the plasma membrane potential (responsible for the general uptake of bioactive molecules), the TPP moiety passes into the mitochondrial matrix. The TPP–antioxidant conjugate localizes in the internal layer of the internal mitochondrial membrane, with the hydrophobic functional group inserted among the lipidic tails and the polar TPP head facing the matrix. Depending on the hydrophobicity and the size of the hydrophobic group, the stability of this position varies.

**Figure 3:**
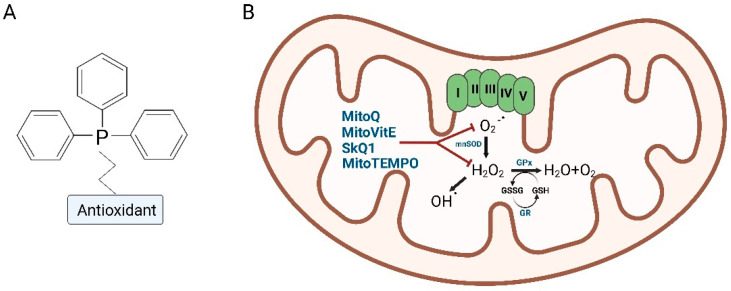
Schematic representation of the TPP–antioxidant conjugate (**A**) and mechanism of action in mitochondria (**B**) (Created with Biorender.com).

In the past decades, many compounds have been linked to TPP to facilitate the mitochondrial uptake, including a number of antioxidant molecules: MitoPBN [77], MitoPeroxidase [78], SkQR1 [79], MitoQ, SkQ1, MitoVitE and MitoTEMPO [71,76,80]. In this review, we specifically focused on four compounds: MitoQ, SkQ1, MitoVitE and MitoTEMPO, since they have been studied and used on cellular and preclinical models of neurodegenerative diseases [71,76,80] (Figure 3 and Table 2).

### 3.1. Mitoquinone (MitoQ)

Mitoquinone (MitoQ) results from the conjugation of the TPP carrier with a modified ubiquinone and is one of the most studied MTAs in the last decade. It was observed that MitoQ had beneficial effects in different animal models of a variety of diseases, accounting for kidney diseases, metabolic syndromes, systemic inflammatory response syndrome, cardiovascular disease, eye diseases, arthritis and age-related pathologies, including neurodegenerative diseases [80,109]. Moreover, together with another TPP-linked antioxidant, SkQ1 (discussed below), MitoQ showed neuroprotective, as well as antiaging, effects.

Considering the promising results in several disease models, researchers launched various clinical trials to measure the potential benefits of MitoQ treatment on neurodegenerative diseases (Table 2). Even though clinical trials on severe diseases failed to replicate the success obtained in the in vivo models, nowadays, MitoQ can be taken as a dietary supplement, since its oral administration has been demonstrated to be tolerable and safe [80,110].

The antioxidant action of MitoQ is linked to its ability to scavenge superoxide, peroxyl and peroxynitrite ROS once it reaches the mitochondria [70,74,80]. Additionally, an interesting factor is its ability to be recycled; oxidized MitoQ can be converted back to active MitoQ (ubiquinol form) by the electron transport chain. In this way, it remains active in the mitochondria, and a continuous low-dose administration allows to reach a steady concentration [32,80].

Preliminary studies in mice assessed the safe dose at 20 mg/(kg of bodyweight) and a significant toxicity at 27 mg/(kg of bodyweight) [75], but there is evidence that a long-term and low-dose MitoQ treatment does not exhibit toxicity in mice, meaning that the toxic effects observed can be traced back to a greater accumulation of TPP in mitochondria that causes a disruption of their normal functions [111].

MitoQ exerted protective activity in in vitro models of Parkinson’s Disease, namely MPTP and 6-OHDA (6-hydroxydopamine hydrochloride)-induced PD models. In SH-SY5Y neuroblastoma cells exposed to 50 mM of 6-OHDA, a reduced mitochondrial fragmentation was observed, together with the reduced activation and translocation of Bax, when MitoQ was administered as a pretreatment [81,112]. Another study evaluated 6-OHDA effects in both a cellular and an animal model at the same time [113]. In N27 cells (rat dopaminergic neuron cell line) treated with MPTP, MitoQ was able to reduce the toxicity, improve the mitochondrial membrane potential and reduce the expression of apoptotic markers. Similarly, in the mouse model exposed to MPTP, MitoQ treatment countered the inactivation of tyrosine hydroxylase, the loss of mitochondrial potential and the activation of apoptotic caspase-3. Furthermore, it was able to improve motor function in the animals [113].

These and other encouraging results prompted researchers to start clinical trials in patients affected by PD. An important, double-blind, 12-month study was carried out on 128 patients that received a dose of 40 or 80 mg per day of MitoQ [32,70,82]. However, the trial was unsuccessful, because MitoQ failed to provide any beneficial effect on PD progression, even at the highest dosage. Probably, a lack of MitoQ efficacy could be explained by poor brain penetration, contrary to predictions, or due to the severity of neuronal damage at the time patients were recruited and treated [32,70,80,82].

Tests on MitoQ were also conducted in Alzheimer’s Disease models, both in vitro and in vivo [32]. In these studies, as in the PD models, the N2a murine neuroblastoma cell line was pretreated with MitoQ and then challenged with amyloid β; the analysis revealed reduced hydrogen peroxide levels, higher ATP levels and improved mitochondrial membrane potential [76,114]. Two in vivo studies used a transgenic mouse model of AD, expressing three human mutant genes: amyloid precursor protein (APP), presenilin-1 (PS-1) and four-repeat tau [93,94]. APP and PS-1 are linked to early-onset forms of human AD, while tau is involved in human frontotemporal dementia. Mice were treated with MitoQ 100 μM or TPP alone. In wild-type mice, the administration had no effect; while in the transgenic model, researchers observed an improved behavioral phenotype and cognitive performance; and in the isolated brains, lower levels of lipid peroxidation (correlated to ROS accumulation), Aβ accumulation and toxicity and caspase activation, with a consequent reduction of synaptic loss.

In a transgenic mouse model of ALS, investigators administered MitoQ 500 μM and analyzed brain, spinal cord and muscular tissues for drug accumulation and disease signs [102]. Muscular tissues were removed, because ALS also involves mitochondria contained in the muscles, as previously shown [47,115,116], making them valuable samples to study mitochondrial alterations. Results from high-resolution respirometry indicated that, after accumulation in the spinal cord and the quadriceps muscle, MitoQ treatment slowed the decline of muscular function. Oxidative stress and pathological signs were markedly reduced when mice received MitoQ; additionally, neuromuscular junctions, together with hindlimb strength, were significantly recovered after treatment. Another interesting observation regarded the ability of MitoQ to prolong the lifespan of transgenic mice [102].

MitoQ was also tested in in vitro and in vivo models of Huntington’s Disease to understand the effects of ROS quenching in this disease. Cultures of striatal neurons expressing mutant HTT (STHDhQ111/Q111) showed characteristic mitochondrial features, such as increased fission and reduced fusion, reduced ATP synthesis and increased oxidative stress [101]. MitoQ administration was able to improve all the parameters taken into consideration: MitoQ-treated cells showed higher cell viability and numbers, a higher activity of mitochondrial fusion proteins than fission proteins, confirmed by the presence of elongated functional mitochondria, and higher ATP yields, together with reduced H_2_O_2_ production and lipid peroxidation. Using the R6/2 mouse model of HD, another study compared wild-type, R6/2 and MitoQ-treated R6/2 mice for various parameters correlated with HD [117]. First, researchers observed reduced weight gain and compromised motor functions in R6/2 mice, which showed a progressive increase of paw clasping scores, lower grasping strength and increased descending time in the vertical pole test but no alterations of locomotion in the open field test. MitoQ treatment was able to improve only the time to descend the pole, with no effect on the other motor tests and weight gain. Investigators wondered whether these results pointed to a possible effect of MitoQ on fine motor control in the R6/2 HD model. This hypothesis seemed to be confirmed by the finding of the highest amount of MitoQ in muscle tissues, while the brain had the lowest amount, indicating a possible low bioavailability in this organ. Coherently, this correlated with lower markers of oxidative stress, particularly evident in muscles, whose marker levels were found to correlate with the results of the vertical pole test: less oxidative stress and damage in muscles, a better result in the motor test (that is, less time to descend the pole). Despite MitoQ not showing effects on the HTT levels in treated mice compared to untreated R6/2 mice, a reduction of autophagy markers LC3-II in the muscles of MitoQ-treated mice was observed, while no effect was observed on LC3-I. These two markers were unaltered in the brain. This study demonstrated that MitoQ treatment of a HD mouse model is more effective on motor functions than on neuronal damage, despite exhibiting the same effects observed in models of other neurodegenerative diseases.

### 3.2. SkQ1

SkQ1 has similar antioxidant activity to MitoQ, but it is composed by the mitochondria targeting molecule TPP conjugated with plastiquinone instead of ubiquinone. Similar to the latter, plastiquinone is able to quench superoxide in mitochondria [32].

Currently, SkQ1 is used in the cosmetic industry, following the publication of a joint study by Russian and Swedish researchers that demonstrated the antiaging properties of this mitochondria-targeted antioxidant. In a mtDNA mutator mouse model, SkQ1 was able to ameliorate the function of mitochondria and cellular respiration, with a consequent weight normalization, improved mobility, delay of age-related pathologies and prolonged lifespan [118]. After this finding, another SkQ1-based drug, Visomitin, already used in Russia as the active principle of eye drops, entered Phase II (NCT02121301) [119] and, shortly thereafter, Phase III (NCT03764735 and NCT04206020) clinical trials in the USA.

The protective effect was demonstrated in cardiomyoblasts when comparing SkQ1 and MitoQ with a classical antioxidant, Vitamin C [120]. TPP-conjugated antioxidants were able, when administered as pretreatment, to reduce ROS and protect from detrimental stimuli, increasing cell viability.

Other in vivo studies are focusing their efforts on neurodegenerative disease models (Table 2).

In 8-week-old mice with MPTP-induced Parkinson’s Disease, SkQ1 was administered daily for one week. After analysis of the striatal tissue, it was observed that SkQ1 had induced higher levels of tyrosine hydroxylase and dopamine, but not those of the DOPA precursor, only in MPTP-treated mice, with no effect in the control mice. These results were confirmed by immunohistochemical analysis of samples from the substantia nigra and ventral tegmental area, which showed increased tyrosine hydroxylase expression and a number of expressing neurons. The authors also evaluated behavioral aspects: motor ability, determined with the open field test, and sensorimotor ability, evaluated with the beam walking test, were significantly impacted in a positive way by SkQ1 administration [83,121].

In a rat model presenting the inherited overproduction of ROS and developing an AD-like pathology (called the OXYS model), researchers introduced SkQ1 in the animal diet; they observed that the antioxidant tended to accumulate in neuronal mitochondria. Moreover, SkQ1 was able to reduce the amyloid β levels and tau hyperphosphorylation while improving memory and learning capabilities, because it allowed to retard mitochondrial dysfunction and structural neurodegenerative alterations [122,123].

### 3.3. MitoVitE

MitoVitE is a mitochondrially targeted Vitamin E also called Mito tacopherol. It presents the same TPP molecule conjugated to α-tocopherol, a type of Vitamin E (E307) and the most potent fat-soluble antioxidant known in nature [124]. Similar to previous TPP-linked antioxidants, it is quickly internalized by cells and subsequently taken up by mitochondria due to its hydrophobicity [76,95].

In the first in vivo studies [75], MitoVitE was demonstrated to accumulate in the heart, brain, muscles, liver and kidneys, more susceptible to mitochondria and oxidative stress.

When first characterized, it was observed that MitoVitE had the ability to counter lipid peroxidation, protecting mitochondria and cells (human osteosarcoma 143B cells and ρ^0^ cells lacking mitochondrial DNA derived from the same cell line) from oxidative damage [125]. In vitro, different groups have further investigated these effects; one study reported that this MTA reduces caspase activation and apoptosis after peroxide exposure, preserving the mitochondrial potential in bovine aortic endothelial cells [126]; another demonstrated that it has a higher efficacy in protecting cells from endogenous oxidative stress and apoptosis than nontargeted antioxidants, as observed in cultured fibroblasts from Friedreich Ataxia patients [127].

However, to this date, the possible application of MitoVitE in neurodegenerative diseases remains to be addressed.

### 3.4. MitoTEMPO

Lastly, MitoTEMPO is another MTA, composed of (2,2,6,6-Tetramethylpiperidin-1-yl)oxyl (TEMPO, piperidine nitroxide) and TPP. Similar to MitoTEMPO is MitoTEMPOL, which presents the nitroxide radical TEMPOL (4-hydroxy-2,2,6,6-tetramethyl- piperidine-1-oxyl). Despite being stable compounds, they present an aminoxyl radical, used to scavenge mitochondrial superoxide; in fact, it is converted into hydroxylamine (radical scavenger), which acts as a SOD mimetic, converting superoxide into water and reducing radicals’ burden [76,95,128,129,130].

In vitro studies have demonstrated the antioxidant activity of MitoTEMPOL, able to rescue mitochondrial dysfunction and oxidative stress in rat liver extracts. However, its efficacy against neurodegeneration is yet to be investigated [130].

On the other hand, MitoTEMPO has already been tested in in vitro and in vivo models of neurodegenerative diseases, mainly PD and AD.

In rat PC12 cells and primary murine neurons, researchers have observed H_2_O_2_ production following exposure to 6-OHDA, MPTP or rotenone, mimicking the oxidative stress that arises in PD neurons. MitoTEMPO was able to reduce H_2_O_2_ production and cell apoptosis, underlining the role of redox imbalance in neurodegeneration and the beneficial effects of targeting mitochondrial ROS [89].

Additionally, the enzyme isocitrate dehydrogenase (IDH2) is involved in the regulation of the redox status and protection against oxidative damage. A study carried out on N2a cells exploited MPTP to induce oxidative damage. IDH2 silencing in the cells significantly increased the markers of oxidative stress and ROS accumulation. In the same study, IDH2 knockout mice were exposed to MPTP, causing a reduction of tyrosine hydroxylase and increase in apoptosis in brain neurons. However, when MitoTEMPO was administered to MPTP-treated mice, it was able to increase the number of tyrosine hydroxylase-expressing neurons [90].

Researchers have studied the impact of MitoTEMPO on primary mouse cortical neurons exposed to amyloid β. MitoTEMPO was demonstrated to be effective in mitigating ROS-induced damage through the reduction of ROS production and Aβ-induced lipid peroxidation, also preventing SOD2 inhibition and mtDNA damage [128].

Another complex study analyzed the aggregation of tau protein oligomers in the brain of human Alzheimer’s Disease patients, tauopathy mice and primary cortical neurons from tau mice. In human AD brains, tau aggregates were more present in the hippocampal region, correlating with an increased ROS accumulation. The same result was obtained in tauopathy mice. Interestingly, when tauopathy mice neurons were cultured in vitro and treated with MitoTEMPO, researchers observed a reduction in tau oligomers, almost reaching the levels of the control samples. This MTA was also able to ameliorate oxidative phosphorylation and mitochondrial defects, lowering ROS levels [98].

MitoTEMPO was also studied in a mouse model carrying the Wobbler mutation, used as a model of ALS. Tissue samples were observed to have higher levels of p53 and ROS, together with lower levels of total glutathione. MitoTEMPO administration in mice resulted in a reduced number of DNA double-stranded breaks observable in dissociated motor neurons, pointing out its protective effect against ROS-induced cell damage [103].

## 4. ROS, Mitochondria and Neurodegenerative Diseases

The pathologic conditions at the mitochondrial level translates in the alteration of cellular homeostasis, with multiple morphofunctional changes, including alterations of mitochondrial membranes and their cristae, the accumulation of mutated mtDNA genes and proteins, decreased ATP and increased ROS production [4,131,132,133,134,135].

In particular, as pointed out by the literature, the role of ROS seems to represent a major trigger that determines the domino effect in the alteration of mitochondrial function [136]. Consequently, the identification of effective antioxidant systems and therapeutics becomes crucial when approaching oxidative stress-induced pathologies.

MTAs have been shown to be able to interrupt the intramitochondrial cascade caused by oxidative damage and leading to cellular apoptosis [70,74,80,89,90,120,125], underlining the importance of addressing the ROS imbalance and its effects on mitochondrial functions.

Oxidative phosphorylation is particularly important and active in neurons because of their role in neurotransmission. The activity level of the respiratory chain rises when the cell takes damage, e.g., at the myelin sheath covering axons, as it occurs in Multiple Sclerosis [137,138,139,140,141,142]. In this context, harmful feedback determines a higher energetic demand, which corresponds to a progressive increase in the ROS levels that cannot be neutralized by physiological antioxidant systems. This buffering system is even more inadequate when a neuroinflammatory process is in progress. However, MTA administration and accumulation in the mitochondria of neurons, as well as of supporting cells (microglia and oligodendrocytes), help to deal with the excessive ROS and restore normal functions [107]; the quenching of ROS reduces microglia activation and tissue inflammation, allowing oligodendrocytes to form new myelin, while ATP production rises in neurons.

In the pathologic context of Alzheimer’s Disease, high concentrations of ROS stimulate neurons to overproduce lipids, which can be attacked by ROS themselves, with the consequent formation of lipid peroxides, detrimental for the cell health [143,144,145,146,147,148]. Various MTAs have demonstrated their effectiveness in restoring neuronal functions in AD models; again, the reduction of ROS from these antioxidants relieves cellular stress and damage, restoring the energy supply and lipids transfer to microglia. If the brain is able to get rid of excessive ROS, it will counter the detrimental effects of Aβ aggregates and also improve their clearance [143,147,149].

Instead, the imbalance between the biogenesis of new mitochondria and the elimination of damaged ones through mitophagy represents a component of undeniable importance in the pathogenesis of Parkinson’s Disease [150,151,152,153,154]. In fact, some causative disease genes identified in the small percentage of hereditary cases (10–15%) encode for proteins involved in the mitophagy process; thus; this may play a key role in PD neurodegeneration, causing the accumulation of defective mitochondria that hamper neurons [155].

Alterations of mitophagy have also been observed in AD, in which Aβ and tau aggregates contribute to the retention of dysfunctional mitochondria: they interfere with both fission regulation and axonal transport, blocking the activities of proteins mediating these processes [156]; thus, they exacerbate the ROS accumulation and oxidative damage.

In Huntington’s Disease, mutant HTT showed effects not only on mitophagy but also on mitochondrial trafficking and dynamics, once again contributing to the accumulation of defective mitochondria and their ROS [19,56,58,62].

MTAs cannot directly target the process of mitophagy and the involved proteins; however, they help the endogenous antioxidant system to deal with the increase of ROS in accumulated defective mitochondria. Doing so, cells do not lose mitochondria; on the contrary, they are able to recover those damaged by the pathology and restore normal function [101,112,114].

This pathological setting is associated with a cytokine profile characterized by an increase in the serum levels of specific categories of cytokines and chemokines involved in the forefront of the pathogenesis of neuroinflammation [157]. Specifically, in neurodegenerative diseases, the converging role played by IL-1β, IL-6 and TNFα is clear, the cytokines mainly implicated in the functional interaction between the immune and nervous systems and in the neuronal dysfunction leading to neurodegeneration [158]. At the neuronal level, these cytokines cause a drop in serotonin, dopamine and adrenaline neurotransmitters that triggers a neuroinflammatory phenotype. Moreover, oxidative stress and consequent mitophagy sustain the neuroinflammation, accompanied by a leakage of mitochondrial DNA (mtDNA) from damaged mitochondria that physiologically tends to gradually increase with age, contributing to the so-called inflammaging [159,160]. In addition, the similarity between mtDNA and bacterial DNA and its oxidized state stimulates the immune response and promotes inflammation. In fact, the inflammatory stimulus of mtDAN is mediated by Toll-Like Receptor 9 (TLR-9) and can involve the NLRP3-inflammasome [161,162] and the pathway of STimulator of INterferon Genes (STING). The activation of NLRP3 induces a switch from pro-IL1β and pro-IL18 to the respective activated forms, IL1β and IL-18, while the activation of IFN-γ is a mechanism known to be associated with autoinflammatory diseases, even those featuring specific neuronal involvement.

## 5. Conclusions

In order to identify, in the most effective way, precise therapeutic targets in the mitochondrial compartment, it would be useful to evaluate the morphological and functional changes of every cellular type that, other than neurons, interacts in the central nervous system, namely astrocytes, oligodendrocytes, pericytes and microglial cells, all contributing to the brain homeostasis. The role of the so-called neurovascular unit [163,164] has been fully recognized in the evaluation of the pathogenesis of neurodegenerative diseases (Figure 2). Nevertheless, at the moment, we still need to clarify its contribution as a pharmacological target in regard to mitochondrial dysfunction.

MTAs exhibited interesting results in disease models, but it seems we cannot optimize them for therapeutic applications in humans yet. The reasons behind this gap are still unclear, and further investigation is needed, possibly taking into consideration the influence of the complex interactions between neurons and supporting cells on the activity of mitochondria-targeted drugs (Table 2).

One of the major issues regarding the effectiveness of MTAs is that damaged mitochondria may have a lower accumulation of these molecules than functional mitochondria, due to a reduced transmembrane potential. This is a critical factor for MTAs localization into these organelles; consequently, they may not exert their function [70,165]. Additionally, one must consider that MTAs are not able to distinguish among healthy and dysfunctional mitochondria; therefore, these molecules could potentially reduce the physiological amount of mitochondrial ROS in healthy tissues, interfering with normal cellular homeostasis [70,165]. Lastly, it is worth remembering that we do not know the pharmacological effects of these compounds, as it has been demonstrated from studies showing the prooxidative properties of some MTAs in specific experimental settings where they promoted cell death, directly leading to negative results in preclinical models [166].

Lastly, the mitochondrial dynamics and mitophagy are appealing targets as well, since many studies have pointed at their central role in the development of neurodegenerative and other diseases [156]. A combined therapeutic approach with antioxidants might further ameliorate the effectiveness and pave the way for clinical applications. In fact, considering the obtained experimental data, it is necessary to develop combined strategies with conventional drugs that might be useful for long-term inhibition of the progression of a specific neurodegenerative disease.

## Figures and Tables

**Table 1:** Mechanisms involved in neurodegenerative diseases.

Neurodegenerative Diseases	Mechanisms Involved	References
Parkinson’s disease (PD)	- Accumulation of intraneuronal Lewy bodies - Interaction of a-synuclein aggregates with transport machineries of the outer membrane - Inhibition of Complex I - Alteration of mitophagy	[32,33,34,35,36,37,38,39,40,41,42]
Alzheimer’s disease (AD)	- Formation of Ab aggregates and tau fibrils - Blocking of transport of mitochondria towards synapses - Increase of mitochondrial fission - Deregulation of mitophagy	[32,34,43,44,45,46]
Amyotrophic lateral sclerosis (ALS)	- Mitochondrial and endoplasmic reticulum abnormalities - Disruption of signaling between mitochondria and endoplasmic reticulum	[47,48,49,50]
Multiple Sclerosis (MS)	- Autoreactivity of CD4+ T helper type 1 cells against myelin components - Overactivation of mitochondrial respiratory chain - Alterations in mitochondrial membranes and mtDNA - Mitochondrial fragmentation - Demyelination	[51,52,53,54,55]
Huntington’s disease (HD)	- Mutation of HTT protein - Induction of higher levels of ROS - Reduced or no activity of Complexes II, III and IV - Increased mitochondria fission - Impaired mitochondria trafficking ad mitophagy	[19,56,57,58,59,60,61,62,63]

**Table 2:** Preclinical and clinical trials testing mitochondria-targeted antioxidants in neurodegenerative diseases.

Neurodegenerative Diseases	MitoQ	MitoTEMPO	MitoVitE	SKQ1
Parkinson’s disease (PD)	[81,82,83,84,85,86,87,88]	[89,90,91,92]	[88,92]	[83,92]
Alzheimer’s disease (AD)	[76,88,93,94,95,96,97]	[76,92,95,98,99]	[76,88,92,95]	[92,100]
Huntington’s disease (HD)	[56,88,101]	[76]	[88]	
Amyotrophic lateral sclerosis (ALS)	[102,88]	[103]	[88]	
Multiple Sclerosis	[104,105]	[106]		[107,108]

## Data Availability

Not applicable.
